# Determinants of Weight Loss following Laparoscopic Sleeve Gastrectomy: The Role of Psychological Burden, Coping Style, and Motivation to Undergo Surgery

**DOI:** 10.1155/2015/626010

**Published:** 2015-11-15

**Authors:** Andrea Figura, Anne Ahnis, Andreas Stengel, Tobias Hofmann, Ulf Elbelt, Jürgen Ordemann, Matthias Rose

**Affiliations:** ^1^Charité Center for Internal Medicine and Dermatology, Division for General Internal and Psychosomatic Medicine, Charité-Universitätsmedizin Berlin, Charitéplatz 1, 10117 Berlin, Germany; ^2^Charité Center for Internal Medicine with Gastroenterology and Nephrology, Division for Endocrinology, Diabetes and Nutrition, Charité-Universitätsmedizin Berlin, Charitéplatz 1, 10117 Berlin, Germany; ^3^Charité Center for Obesity and Metabolic Surgery, Charité-Universitätsmedizin Berlin, Charitéplatz 1, 10117 Berlin, Germany

## Abstract

*Background*. The amount of excess weight loss (%EWL) among obese patients after bariatric surgery varies greatly. However, reliable predictors have not been established yet. The present study evaluated the preoperative psychological burden, coping style, and motivation to lose weight as factors determining postoperative treatment success.* Methods*. The sample included 64 morbidly obese patients with a preoperative BMI of 51 ± 8 kg/m^2^ who had undergone laparoscopic sleeve gastrectomy (LSG). Well-established questionnaires were applied before surgery to assess the psychological burden in terms of “perceived stress” (PSQ-20), “depression” (PHQ-9), “anxiety” (GAD-7), and “mental impairment” (ISR) as well as coping style (Brief COPE) and motivation to lose weight. %EWL as an indicator for treatment success was assessed on average 20 months after surgery.* Results*. Based on the %EWL distribution, patients were classified into three %EWL groups: low (14–39%), moderate (40–59%), and high (60–115%). LSG patients with high %EWL reported significantly more “active coping” behavior prior to surgery than patients with moderate and low %EWL. Patients' preoperative psychological burden and motivation to lose weight were not associated with %EWL.* Conclusion*. An “active coping” style might be of predictive value for better weight loss outcomes in patients following LSG intervention.

## 1. Introduction

Obesity has become an increasingly important global health problem. Currently, 13% of the world's adult population aged 18 years and older are obese, with a body mass index (BMI) ≥ 30 kg/m^2^ [1]. In 2008–2011, 23% of the male and 24% of the female adult population in Germany had a BMI ≥ 30 kg/m^2^ [2]. Bariatric surgery is the most effective long-term treatment for the majority of morbidly obese patients with a BMI ≥ 40 kg/m^2^ or for those with a BMI ≥ 35 kg/m^2^ who are diagnosed with obesity-related medical comorbidities such as type 2 diabetes mellitus or arterial hypertension [3].

In previous studies of weight loss outcomes after bariatric surgery, the amount of excess weight loss as a percentage (%EWL) was commonly used as a marker of weight loss success (EWL ≥ 50%) or weight loss failure (EWL < 50%) [4, 5]. %EWL is calculated using the following formula: (postoperative weight loss)/(preoperative excess weight) × 100. BMI ≥ 25 kg/m^2^ is recognized as the lowest limit of overweight, and therefore excess weight is calculated relative to a BMI of 25 kg/m^2^ [6, 7]. However, the dichotomous classification into success* versus* failure, with an arbitrarily defined cut-off at 50% EWL, does not reflect the considerably wide individual variation in postoperative weight loss that has been described in previous studies [8, 9]. Approximately 15% to 20% of all bariatric surgery patients fail to achieve adequate %EWL [10, 11]; inadequate weight loss is considered to be EWL < 25% according to the Reinhold criteria [12]. Surprisingly little is known about the factors that promote or hinder weight loss after bariatric surgery.

Given the background of a multifactorial etiology of obesity, a multidisciplinary evaluation before bariatric surgery is recommended, and the assessment of psychosocial factors in addition to the medical examination has become highly relevant [13, 14]. However, although the number of bariatric procedures performed is rising [15, 16], conclusive empirical evidence about the impact of psychosocial factors on postoperative weight loss outcomes is still lacking.

Psychological factors and mental disorders have been associated with weight loss results after bariatric surgery. Notably, up to 70% of obese patients considering bariatric surgery present with high rates of mental comorbidities, with depression, anxiety, and eating disorders being the most prevalent [17, 18]. Within the current medical-theoretical framework, causal pathways between obesity and mental disorders are likely to be bidirectional. On one hand, being a target of weight-based discrimination and stigmatization might lead to depressive symptoms with feelings of worthlessness, social anxiety, and isolation, especially in extremely obese patients [19, 20]. On the other hand, depression and anxiety disorders might contribute to weight gain by interfering with healthy eating behaviors and might complicate weight loss [21]. Eating in response to negative emotions might have an anxiolytic effect, and an increased appetite extending to overeating can be a symptom of depression. Furthermore, stress might play an important intermediating role in the association between obesity and mental disorders: under conditions of chronic stress, the activity of the hypothalamic-pituitary-adrenal axis, which responds to stress by releasing cortisol and hormones that modulate sympathetic nervous system activity, becomes dysregulated, a state that has been implicated in depression and anxiety disorders as well as in obesity [22].

Obese patients with comorbid mental disorders might have difficulties in adhering to the behavioral changes required to benefit from bariatric surgery [23]. However, the predictive value of preoperative depression and anxiety disorder for weight loss outcomes after bariatric surgery is controversial; different studies have shown a negative influence [24], a positive influence [25], or no effect [26]. Additionally, there is evidence that the severity rather than the types of mental disorders appears to be more relevant for weight loss outcomes; that is, a greater overall psychological burden is associated with less weight loss after bariatric surgery [27, 28].

Coping strategies, used when confronting difficult situations in daily life, and the motivation to undergo surgery might also be related to different weight loss outcomes; however, research is scarce. Recent studies have reported more avoidant and (delegated) active coping in patients seeking bariatric surgery* versus* patients seeking conservative treatment options [29] or more avoidance and depressive coping reactions in a subgroup of emotional dysregulated/undercontrolled* versus* resilient/high-functioning prebariatric women [30]. Although maladaptive coping behavior might complicate postbariatric weight loss, clear associations have not been established yet.

To date, most research on the role of prebariatric psychological variables in treatment success has focused on gastric bypass surgery. Laparoscopic sleeve gastrectomy (LSG) as a restrictive single-stage procedure is relatively new in the field of bariatric surgery but has already proven its efficacy in weight reduction with low surgical risks [31]. To our knowledge, no studies have systematically assessed the relationship between postoperative %EWL and preoperative psychological burden (as a broader construct comprising “perceived stress,” “depression,” “anxiety,” and “mental impairment”), coping style, and the motivation to undergo surgery in a clinical sample of LSG patients.

Hence, assessing a broad range of preoperative patient characteristics might be useful to identify homogeneous subgroups of LSG patients with different needs to tailor interventions and optimize postoperative weight loss outcomes and well-being. Therefore, the main aim of the present study was to characterize patients with low, moderate, and high postoperative %EWL retrospectively using between-group comparisons to examine whether LSG patients with different %EWL levels after surgery differed preoperatively in (1) their psychological burden in terms of the levels of “perceived stress,” “depression,” “anxiety,” and “mental impairment,” (2) coping style (e.g., “active coping”), and (3) their motivation to lose weight. As a secondary aim, we wanted to study whether the %EWL groups differed on a range of preoperative patient characteristics, such as (a) weight and BMI, (b) sociodemographic status, (c) clinically diagnosed comorbidities including eating disorders, and (d) the use of psychotherapy. To do so, LSG patients were evaluated approximately 12 months before surgery and on average 20 months after surgery.

## 2. Subjects, Materials, and Methods

### 2.1. Study Design and Participants

As part of the presurgical psychosomatic evaluation for bariatric intervention at the Multidisciplinary Obesity Center of the University Hospital Charité-Universitätsmedizin Berlin in Germany, patients who planned to undergo bariatric surgery were assessed by an experienced clinical psychologist or physician specialized in psychosomatic medicine. A semistructured interview was performed for the psychosocial assessment and diagnosis of mental disorders including eating disorders according to the International Classification of Diseases (ICD-10) [32]. Additionally, tablet PCs were used to obtain psychometric measurements of psychological variables (i.e., the severity of “perceived stress,” “depression,” “anxiety,” and “mental impairment” as well as “coping style” and “motivation to lose weight”) by employing well-established standard questionnaires. In accordance with the German Guidelines for Obesity Surgery [14, 33], bariatric surgery was not offered to patients with mental retardation or severe untreated psychiatric disorders such as schizophrenia, emotionally unstable personality disorder, alcohol or drug abuse/dependence, and suicidality. Further medical consultations involved a surgeon and an endocrinologist.

Between April 2009 and August 2012, a medical database was used to identify 96 bariatric surgery patients who underwent LSG and fully completed the presurgical psychometric assessment, and they were contacted for follow-up. From that sample, 71% (*n* = 68) of the patients participated in the follow-up, and 29% (*n* = 28) of them declined participation or could not be reached. For the present study, we excluded patients with less than 1 year of follow-up after LSG surgery (*n* = 4). Moreover, bariatric surgery patients who underwent gastric banding or Roux-en-Y gastric bypass surgery were not included in our study because we sought to assess a homogeneous sample and to eliminate the effect of type of surgical procedure on %EWL (due to the significantly inferior/superior weight loss achievable by these procedures [34]).

The present cohort consisted of 64 morbidly obese patients (46 women and 18 men) who had undergone LSG. The average points of assessment were 12 months prior to surgery and 20 months after surgery. The study was approved by the local Institutional Review Board. Written informed consent was obtained from all participants included in the study.

### 2.2. Materials and Measures

Data collected preoperatively included sociodemographic information, clinically diagnosed comorbidities (metabolic syndrome, mental disorders including eating disorders such as binge eating disorder, night eating syndrome, and sweet eating syndrome, or disordered eating behaviors such as hyperphagia, which describes a subsyndromal excessive eating behavior and/or an increased high-calorie food intake similar to terms such as emotional overeating and grazing without reaching the criteria for binge eating disorder), and the use of past or current psychotherapy. For the psychometric assessment of psychological variables, the following validated (with the exception of the “motivation to lose weight” survey) self-rating questionnaires were employed* via* tablet PCs.

*Stress* was assessed using the Perceived Stress Questionnaire (PSQ) [35] in its revised German 20-item version (PSQ-20) [36]. The instrument assesses subjectively experienced stress reactions (worries, tension, and joy) and the perception of nonspecific external stressors (demands). An overall index score is calculated from all items with values between 0 and 1; higher scores indicate higher levels of perceived stress. The internal consistency of the PSQ-20 total score was indicated by Cronbach's *α* = 0.94.

*Depression* was assessed using the German version of the Patient Health Questionnaire Depression Scale (PHQ-9) [37, 38]. PHQ-9 is a 9-item screening instrument for the diagnosis of major depression and the assessment of depressive symptom severity. The total score ranges from 0 to 27. The internal consistency was represented by Cronbach's *α* = 0.87, and the level of agreement between the clinical diagnosis of depression according to ICD-10 and the PHQ-9 score (cut-off ≥ 10) was moderate (Cohen's *k* = 0.45); specificity was 84%, and sensitivity was 64%.

*Anxiety* was assessed using the German version of the Generalized Anxiety Disorder 7-item (GAD-7) scale [39, 40]. GAD-7 is a brief screening instrument for the diagnosis of generalized anxiety disorder and the assessment of symptom severity. The total score ranges from 0 to 21, and the cut-off score is 10. The internal consistency was indicated by Cronbach's *α* = 0.88.

*Mental impairment* was assessed using the ICD-10 Symptom Rating (ISR) [41]. This 29-item instrument assesses psychological syndromes and mental disorders according to the ICD-10. The ISR total score assesses the overall severity of the patient's mental impairment. Scores range from 0 to 3, with higher scores indicating higher levels of mental impairment. The internal consistency of the ISR total score was indicated by Cronbach's *α* = 0.92.

*Coping style* was assessed using the German version of the Brief COPE questionnaire [42, 43]. Patients were asked to think of their usual thoughts and actions when facing a difficult situation. The Brief COPE consists of 28 items that assess four coping styles: “avoidant coping” (i.e., self-blame, denial, and venting), “seeking support” (i.e., the use of instrumental support, emotional support, and religion), “positive reframing” (i.e., acceptance, positive reframing, and humor), and “active coping” (i.e., active coping and planning). Scores range from 1 to 4, with higher scores indicating higher levels of coping on each scale. The levels of internal consistencies were indicated by Cronbach's *α* = 0.38–0.85.

*Motivation to lose weight* was assessed with specific* ad hoc* questions. A motivation survey was developed with 10 items asking how strongly patients were “self-motivated” to lose weight or motivated by their “social environment” (i.e., partner, family/children, friends, colleagues, and employer) or “treatment environment” (i.e., physician, health insurance, nutritionist, and therapist). Patients indicated their answers on a 5-point scale that ranged from 1 =* not at all* to 5 =* very strong*. The levels of internal consistencies were indicated by Cronbach's *α* = 0.79–0.84.

Body weight (kg), height (cm), and BMI (kg/m^2^) were extracted from the medical database. On average, 20 months after surgery, weight status was either extracted from the medical database or self-reported, and weight loss was assessed. As a standard for evaluation, weight change is reported as the percentage of excess weight loss (%EWL), which is calculated using the following formula: (postoperative weight loss)/(preoperative excess weight at time of surgery) × 100. BMI ≥ 25 kg/m^2^ is recognized as the lowest limit of overweight, and therefore excess weight is calculated in relation to a BMI of 25 kg/m^2^ [6, 7]. %EWL of 100% indicates the achievement of normal weight.

### 2.3. Statistical Analyses

All analyses were conducted using SPSS version 22 (IBM, Armonk, NY), and the alpha level of statistical significance was set at *p* < 0.05. Patients were classified into three groups of nearly equal size according to the observed postoperative %EWL distribution of the sample based on tertiles. One-way between-groups multivariate ANOVAs were performed to compare patients with low, moderate, and high %EWL (= independent variable) with respect to group differences in preoperative (1) psychological burden, (2) coping style, and (3) motivation to lose weight (= dependent variables). An exploratory principal component analysis showed high correlations among “perceived stress” (PSQ-20), “depression” (PHQ-9), “anxiety” (GAD-7), and “mental impairment” (ISR) (*r* = 0.78–0.89), and strong loadings (>0.90) of each of these variables on a single factor explained 87% of the total variance in the data set. Therefore, in accordance with the constructs “coping style” and “motivation to lose weight,” the broader construct “psychological burden” was generated and used for further multivariate analyses to retain statistical power. For the combined dependent variable “psychological burden,” four variables were used: “perceived stress” (PSQ-20), “depression” (PHQ-9), “anxiety” (GAD-7), and “mental impairment” (ISR). For the combined dependent variable “coping style,” four variables of Brief COPE were used: “avoidant coping,” “seeking support,” “positive reframing,” and “active coping.” For the combined dependent variable “motivation to lose weight,” three variables were used: “self-motivation,” “social environment,” and “treatment environment.” For additional exploratory analyses, univariate ANOVAs were performed with Bonferroni-adjusted* post hoc* comparisons using Tukey's HSD test to control for Type I error. Chi-square tests were conducted for the nominal dependent variables, with* post hoc* comparisons using standardized residuals and a critical value of ±1.96 indicating significant group differences. The magnitude of group differences was further analyzed by means of effect sizes; for metrical data, we used Cohen's *d* (0.2 = small, 0.5 = moderate, and 0.8 = large), and for nominal data, we used Cohen's *w* (0.1 = small, 0.3 = moderate, and 0.5 = large). For correlative analyses, Pearson's product-moment correlation coefficient *r* was used. Preliminary analyses included describing the variables, screening for missing values and outliers, and testing for the normality, linearity, homogeneity of variance/variance-covariance matrices, and multicollinearity of the dependent variables, with no serious violations noted.

## 3. Results

### 3.1. %EWL Groups

The mean postoperative %EWL was 53% (SD = 24%, range: 14–115%). In relation to the observed %EWL distribution in our study, patients were classified into three %EWL groups of nearly equal size based on tertiles: low (*n* = 21, EWL range: 14–39%), moderate (*n* = 22, EWL range: 40–59%), and high (*n* = 21, EWL range: 60–115%). Postoperative %EWL differed significantly between the three %EWL groups (*p* < 0.001). The results showed no significant group difference in the follow-up time interval (*p* > 0.05) (Table 1).

### 3.2. Weight and BMI

Weight and BMI characteristics for the low-%EWL, moderate-%EWL, and high-%EWL groups before and after bariatric surgery are presented in Table 1. Within all three %EWL groups, the patients' mean weight, excess weight, and BMI decreased significantly after surgery (*p* < 0.001). Significant preoperative group differences between patients of moderate and high %EWL were detected: patients with high %EWL had a lower preoperative weight (*p* = 0.018), excess weight (*p* = 0.012), and BMI (*p* = 0.015). Preoperative weight and BMI differences between the low-%EWL and moderate-%EWL groups and between the low-%EWL and high-%EWL groups did not reach statistical significance (*p* > 0.05). In the high-%EWL group, 38% (*n* = 8) of the patients attained a postoperative nonobese BMI of <30 kg/m^2^.

### 3.3. Sociodemographic Status

Preoperative sociodemographic characteristics for the groups with low, moderate, and high %EWL are presented in Table 2. Among the three %EWL groups, there were no significant differences in age, sex, partnership, or employment status before surgery (*p* > 0.05). A small but statistically significant group difference in education levels was found: on average, the high-%EWL group had 2.2 more years of education than the low-%EWL group (*p* = 0.010). There were no statistically significant differences in education levels between the low-%EWL and moderate-%EWL groups or between the moderate-%EWL and high-%EWL groups (*p* > 0.05).

### 3.4. Comorbidities and Use of Psychotherapy

With regard to physical comorbidities, Table 2 shows that all three %EWL groups had high rates of presurgically diagnosed metabolic syndrome symptoms; approximately half of the patients in each group suffered from type 2 diabetes mellitus. No group differences in diabetes prevalence were found (*p* > 0.05). With regard to mental comorbidities, the groups with low and high %EWL presented with particularly high rates of presurgically diagnosed mental disorders (i.e., depression); however, the results showed no statistically significant group differences in the prevalence of mental disorders (*p* > 0.05) (Table 2). All three %EWL groups presented with high rates of disordered eating behaviors such as hyperphagia. Group differences in the prevalence of eating disorders such as binge eating disorder, night eating syndrome, or sweet eating syndrome could not be detected (*p* > 0.05) (Table 2). Altogether, a total of 39% (*n* = 25) of the patients reported past or current psychotherapeutic treatment. The results showed a statistically significant group difference in the use of psychotherapy: slightly more patients in the low-%EWL group underwent psychotherapeutic treatment before LSG surgery compared with those in the moderate-%EWL group (*p* = 0.013). For the high-%EWL group, no statistically significant difference in the use of psychotherapy could be identified (*p* > 0.05) (Table 2).

### 3.5. Psychological Burden, Coping Style, and Motivation to Lose Weight

Preoperative psychological characteristics for the groups with low, moderate, and high %EWL are presented in Table 2. The results revealed no statistically significant difference between the three %EWL groups in the overall preoperative psychological burden in terms of “perceived stress” (PSQ-20), “depression” (PHQ-9), “anxiety” (GAD-7), and “mental impairment” (ISR) (*F*_(8,118)_ = 0.9, *p* = 0.535; Pillai's trace = 0.1; *d* = 0.5). Furthermore, the three %EWL groups showed no statistically significant difference in their overall preoperative motivation to lose weight in terms of “self-motivation” or motivation from the “social environment” or “treatment environment” (*F*_(6,118)_ = 1.0, *p* = 0.428; Pillai's trace = 0.1; *d* = 0.4).

**Figure 1:**
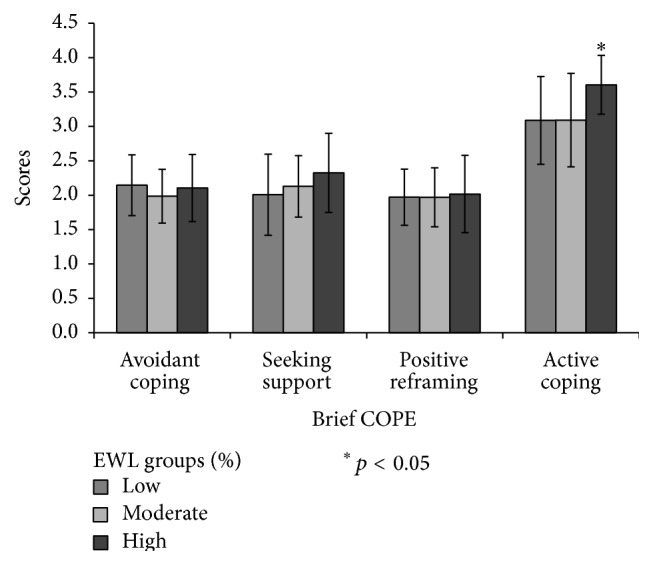
Preoperative coping style according to postoperative %EWL groups. Brief COPE scores before LSG for the groups with low, moderate, and high postoperative %EWL. Bars represent the standard deviation. The asterisk indicates statistically significant group differences between the high-%EWL and low-%EWL groups and the high-%EWL and moderate-%EWL groups.

In terms of the overall preoperative coping style (Brief COPE), the three %EWL groups showed no statistically significant difference (*F*_(8,118)_ = 1.6, *p* = 0.145; Pillai's trace = 0.2; *d* = 0.7). To further investigate our main research question (2), exploratory* post hoc* analyses were conducted. When the results for the different coping styles (“avoidant coping,” “seeking support,” “positive reframing,” and “active coping”) were considered separately in Bonferroni-adjusted comparisons, a marked difference could be identified: patients with high postoperative %EWL reported slightly but significantly higher scores for “active coping” behavior prior to surgery compared with patients with low %EWL (*p* = 0.019) and moderate %EWL (*p* = 0.022). The low-%EWL and moderate-%EWL groups did not differ in this regard (*p* > 0.05) (Table 2 and Figure 1). The positive relationship between preoperative “active coping” and postoperative %EWL was confirmed by a statistically significant correlation analysis (*r* = 0.36; *p* = 0.004).

### 3.6. Attrition Analysis

The results showed no statistically significant differences between patients who dropped out of the study (*N* = 28) and patients who were reassessed after LSG (*N* = 64) in preoperative variables such as age, sex, BMI, years of education, prevalence of metabolic syndrome symptoms, mental disorders including eating disorders, or psychotherapy use (*p* > 0.05). Furthermore, the two groups showed no statistically significant differences in their overall preoperative psychological burden (*F*_(4,87)_ = 1.9, *p* > 0.05; Pillai's trace = 0.08; *d* = 0.6), coping style (*F*_(4,87)_ = 1.0, *p* > 0.05; Pillai's trace = 0.04; *d* = 0.4), or motivation to lose weight (*F*_(3,87)_ = 2.1, *p* > 0.05; Pillai's trace = 0.07; *d* = 0.5) (data not shown).

## 4. Discussion

In the present study, we employed a retrospective approach to examine associations between preoperative factors and the amount of %EWL achieved on average 20 months after bariatric surgery in 64 patients who underwent LSG. Patients were classified into groups with low, moderate, and high postoperative %EWL based on tertiles to identify homogeneous subgroups of LSG patients for group comparisons.

Our results showed that LSG patients with high %EWL had a lower weight and BMI prior to surgery compared with patients with moderate %EWL, but no significant weight and BMI differences compared with patients with low %EWL were found. This initially counterintuitive finding is in line with previous studies of gastric bypass surgery reporting that patients who were preoperatively less obese have a higher postoperative %EWL [9, 44, 45]. One possible explanation is that patients who were preoperatively more obese (i.e., BMI ≥ 50 kg/m^2^) tend to stagnate earlier in their weight loss process and begin to regain weight as early as 12 months after surgery [9]. Although early weight stagnation and weight regain might play a role, our prepost study design masks the time course of weight change, and therefore explanations remain speculative at this point. Longitudinal data are necessary to assess the course of weight after LSG in more detail.

The preoperative psychological burden and motivation to lose weight were not associated with the amount of %EWL in patients following LSG intervention. However, more self-reported “active coping” behavior (assessed by the following items: “I have been concentrating my efforts on doing something about the situation I am in”; “I have been taking action to try to make the situation better”; “I have been trying to come up with a strategy about what to do”; and “I have been thinking hard about what steps to take”) seemed to be beneficial for treatment success in terms of more favorable weight loss outcomes. In summary, a more “active coping” style might be predictive of higher postoperative weight loss outcomes. However, the results provided no clear evidence of a clinically important role of preoperative mental health and the motivation to lose weight in the weight-related treatment success of LSG.

Previous studies have identified psychological predictors of weight loss outcomes after bariatric surgery; for example, some have observed less weight loss in patients with mood and anxiety disorders [24, 27]. However, across reviews of the prebariatric predictors of postbariatric weight loss [28, 46], relationships between psychological factors and weight-related surgery outcomes seem inconsistent and appear to vary by study design and sample. On one hand, obese patients receiving bariatric surgery constitute a highly selective group. Bariatric surgery patients must meet certain criteria to be eligible for surgery (e.g., they must meet a certain BMI requirement, be free of potential contraindications, and obtain approval from health care team members), which evidently results in a homogenized sample of medically and mentally healthier patients. Therefore, the variability within our sample of patients who underwent LSG surgery might be reduced, which could in turn lower the magnitude of %EWL group differences and underestimate the impact of psychological factors on weight loss outcomes [47]. On the other hand, static factors such as sociodemographic status assessed prior to bariatric intervention might have limited practical value for predicting weight loss because they do not capture patients' flexibility in adapting to changing circumstances after the intervention. In fact, some studies suggest that postbariatric factors such as the (re)occurrence of depressive and anxiety disorders [27, 48] or the loss of control over eating [49] have a much stronger impact on weight loss outcomes after bariatric surgery than prebariatric factors do. How might those possibilities be applicable to our findings? While suggesting tailored interventions to optimize treatment success, our results demonstrate the difficulty of preoperatively identifying patients who are at risk for more unfavorable postoperative weight-related outcomes. However, our results might also indicate that more active coping behavior is a marker of higher postoperative weight loss. As discussed by Ahnis et al. [29], active coping behavior in obese patients seeking a bariatric intervention might include the preoperative search for information about surgical treatment options on the internet, in support groups, in informative meetings, or during medical visits and examinations. This behavior may in turn contribute to patients developing a more informed and educated perspective on the possibilities and limitations of LSG surgery and the necessary lifestyle modifications (e.g., following dietary advice and physical activity recommendations) to achieve higher and sustained weight loss after surgery.

However, because bariatric surgery leads to a profound change in patients' gastrointestinal and whole-body physiology, it is important to also consider potential physiological mechanisms [50]. In fact, such considerations might be complex, and psychological and physiological factors might actually combine and interact in influencing outcomes.

### 4.1. Strengths and Limitations

The strength of our study is that it was conducted in a naturalistic clinical setting. In addition, whereas the majority of previous research in this area focused on Roux-en-Y gastric bypass surgery, we had access to a homogeneous clinical sample of patients undergoing LSG. To the best of our knowledge, this was the first study that systematically assessed the relationship between postoperative %EWL on average 20 months after surgery and the preoperative psychological burden (as a broader construct comprising “perceived stress,” “depression,” “anxiety,” and “mental impairment”), coping style, and motivation to undergo surgery in a sample of LSG patients. The size of our sample was comparable to that of other small-scale studies in the field of bariatric surgery. Moreover, the attrition rate in our study was rather small, and our attrition analyses showed no significant differences in preoperative variables between patients who provided data at follow-up compared with those who did not. However, from a statistical perspective, the sample size is still small, allowing for only a few statistical tests in addition to a large set of exploratory analyses. The patients' postoperative weight was self-reported, which might undermine the validity of the %EWL classification scheme. However, self-reporting of weight is relatively common in obesity studies, and there is evidence that objectively measured and self-reported weights are not significantly different in bariatric surgery patients [49]. Finally, it can be argued that the generation of “psychological burden” as a broader construct comprising “perceived stress,” “depression,” “anxiety,” and “mental impairment” might be questionable. However, all these variables proved to be strong determinants of patients' psychological burden, with high intercorrelations and high loadings on a single factor, thus supporting the use of the combined variable to capture the construct of psychological burden.

## 5. Conclusion

Although an “active coping” style seemed to be of value in predicting more favorable postoperative weight loss outcomes, neither biomedical markers nor an extensive set of other psychological constructs assessed before surgery enabled a clinically relevant prediction of the weight-related treatment success of LSG. Our study highlights the need for further research on the psychological correlates of postbariatric health outcomes. Nevertheless, despite our finding of a considerably large degree of variation in postoperative weight loss in our sample of LSG patients, all three %EWL groups showed significant weight loss and decreases in body weight and BMI, including the low-%EWL group. Therefore, the bariatric intervention proved to be highly beneficial for the vast majority of patients, thus promoting positive health outcomes.

## Figures and Tables

**Table 1:** Weight and BMI characteristics for groups with low, moderate, and high %EWL before and after laparoscopic sleeve gastrectomy (LSG).

	Postoperative excess weight loss (EWL) in %					
	Group 1	Group 2	Group 3			
	Low %EWL	Moderate %EWL	High %EWL			
*N* = 64	*n* = 21	*n* = 22	*n* = 21			

	M (SD)	M (SD)	M (SD)	*F*	*p*	*d*

%EWL Range	30.3 (7.5) 14–39	48.6 (6.0) 40–59	80.9 (17.8) 60–115	**102.0**	**<0.001**	**3.7**

Follow-up (months)	19.9 (5.4)	18.5 (5.9)	20.9 (8.7)	0.7	0.511	0.3
Weight (kg)						
preOP	154.1 (24.7)	161.7 (37.9)	136.3 (23.7)	**4.1**	**0.021**	**0.7**
postOP	129.9 (18.5)	118.9 (23.3)	84.3 (13.7)	**33.0**	**<0.001**	**2.1**
Excess weight (kg)						
preOP	79.9 (21.4)	87.6 (31.2)	64.9 (21.4)	**4.5**	** 0.015**	**0.8**
postOP	55.7 (15.7)	44.8 (15.9)	13.0 (12.1)	**47.9**	**<0.001**	**2.5**
BMI (kg/m^2^)						
preOP	52.1 (7.2)	54.5 (8.5)	47.7 (7.1)	**4.2**	**0.019**	**0.7**
postOP	43.9 (5.5)	40.1 (4.5)	29.6 (4.2)	**51.7**	**<0.001**	**2.6**

Note: preOP, preoperative; postOP, postoperative; %EWL, excess weight loss in percentage; BMI, body mass index. Statistically significant values are marked in boldface.

**Table 2:** Sociodemographics, comorbidities, and psychological characteristics before laparoscopic sleeve gastrectomy (LSG).

	Postoperative excess weight loss (EWL) in %					
	Group 1	Group 2	Group 3			
	Low %EWL	Moderate %EWL	High %EWL			
*N* = 64	*n* = 21	*n* = 22	*n* = 21			

	M (SD)	M (SD)	M (SD)	*F*/*χ* ^2^	*p*	*d*/*w*

%EWL Range	30.3 (7.5) 14–39	48.6 (6.0) 40–59	80.9 (17.8) 60–115			

*Preoperative*						
Sociodemographics						
Age in years	48.4 (11.7)	44.7 (11.5)	43.6 (8.8)	1.2	0.324	0.4
Female sex *n* (%)	13 (61.9%)	15 (68.2%)	18 (85.7%)	3.2	0.205	0.2
Years of education	12.5 (2.3)	13.2 (2.6)	14.7 (2.0)	**4.9**	**0.011**	**0.8**
In partnership *n* (%)	12 (57.1%)	16 (72.7%)	17 (81.0%)	2.9	0.229	0.2
Employed *n* (%)	11 (52.4%)	9 (40.9%)	13 (61.9%)	1.9	0.386	0.2
Comorbidities, clinical diagnosis^a^						
Metabolic syndrome^b^ *n* (%)	18 (85.7%)	20 (90.9%)	18 (85.7%)		—^i^	
Type 2 diabetes mellitus *n* (%)	9 (42.9%)	13 (59.1%)	9 (42.9%)	1.5	0.467	0.2
Mental disorder^c^ *n* (%)	9 (42.9%)	5 (22.7%)	9 (42.9%)	2.5	0.281	0.2
Depression *n* (%)	7 (33.3%)	2 (9.1%)	5 (23.8%)	3.8	0.152	0.2
Eating disorder^d^ *n* (%)	21 (100.0%)	21 (95.5%)	20 (95.2%)		—^i^	
Hyperphagia^e^ *n* (%)	8 (38.1%)	15 (68.2%)	14 (66.7%)	5.0	0.082	0.3
Binge eating disorder^f^ *n* (%)	3 (14.3%)	1 (4.5%)	2 (9.5%)		—^i^	
Psychotherapy^g^ *n* (%)	13 (61.9%)	4 (18.2%)	8 (38.1%)	**8.6**	**0.013**	**0.4**
Psychological variables^h^						
*Psychological burden*						
Perceived stress (PSQ-20)	0.5 (0.2)	0.4 (0.2)	0.5 (0.2)	1.1	0.337	0.4
Depression (PHQ-9)	8.8 (5.7)	6.5 (5.2)	8.8 (5.4)	1.3	0.272	0.4
Anxiety (GAD-7)	8.8 (5.4)	5.5 (5.1)	7.3 (5.4)	2.1	0.137	0.5
Mental impairment (ISR)	1.1 (0.6)	0.8 (0.4)	1.1 (0.7)	1.9	0.162	0.5
*Coping style *(Brief COPE)						
Avoidant coping	2.1 (0.4)	2.0 (0.4)	2.1 (0.5)	0.8	0.456	0.3
Seeking support	2.0 (0.6)	2.1 (0.4)	2.3 (0.6)	1.8	0.173	0.5
Positive reframing	2.0 (0.4)	2.0 (0.4)	2.0 (0.6)	0.1	0.934	0.1
Active coping	3.1 (0.6)	3.1 (0.7)	3.6 (0.4)	**5.0**	**0.010**	**0.8**
*Motivation to lose weight*						
Social environment	2.2 (1.1)	2.3 (0.8)	2.4 (1.1)^j^	0.3	0.752	0.2
Treatment environment	2.4 (1.0)	1.9 (0.6)	2.2 (1.0)^j^	1.9	0.163	0.5
Self-motivation	4.2 (1.2)	4.6 (0.7)	4.6 (0.9)^j^	0.9	0.423	0.3

*Note*. Univariate *F*-test statistics are shown. Statistically significant values are marked in boldface.

^a^Diagnosis made by an experienced clinical psychologist or physician specialized in psychosomatic medicine according to the International Classification of Diseases (ICD-10, WHO, 2006, 2010).

^b^Disorders belonging to the metabolic syndrome along with obesity (*n* = 64) included type 2 diabetes mellitus (*n* = 31), high blood pressure (*n* = 42), dyslipidemia (*n* = 19), and hyperuricemia (*n* = 5).

^c^Mental disorders included depression (*n* = 14), reaction to severe stress and adjustment disorder (*n* = 6), anxiety (*n* = 2), somatoform disorder (*n* = 1), and bipolar disorder (*n* = 1).

^d^Eating disorders included hyperphagia (*n* = 37), binge eating disorder (*n* = 6), night eating syndrome (*n* = 1), sweet eating syndrome (*n* = 1), and eating disorders not otherwise specified (ED-NOS) (*n* = 53).

^e^Hyperphagia is a subsyndromal excessive eating behavior and/or increased high-calorie food intake.

^f^Binge eating is the regular occurrence of eating binges with a feeling of loss of control over eating without compensatory behaviors.

^g^Psychotherapy refers to past or current preoperative mental health treatment by a psychiatrist and/or psychologist.

^h^Psychometric measurements employing tablet PCs.

^i^Due to ceiling effects in our studied sample of obese patients undergoing bariatric surgery not analyzed statistically; minimum expected cell frequency <5.

^j^ *n* = 20.
